# Supplementary material for: Herbaceous perennial plants with short generation time have stronger responses to climate anomalies than those with longer generation time
Source: Nat Commun. 2021 Mar 23;12:1824. doi: 10.1038/s41467-021-21977-9 (PMC7988175; doi:10.1038/s41467-021-21977-9)
Supplement: Supplementary file 1 — Supplementary Information [file 41467_2021_21977_MOESM1_ESM.pdf]

Supplementary information for:

Herbaceous perennial plants with short generation time have stronger responses to climate anomalies than those with longer generation time

Aldo Compagnoni[1,2,*], Sam Levin[1,2], Dylan Z. Childs[3], Stan Harpole[4,2,1], Maria Paniw[5], Gesa Römer[6,7], Jean H. Burns[8], Judy Che-Castaldo[9], Nadja Rüger[2,10,11], Georges Kunstler[12], Joanne M. Bennett[1,2,13], C. Ruth Archer[14,15], Owen R. Jones[6,7], Roberto Salguero-Gómez[16,**] & Tiffany M. Knight[1,17,2**]

[1] Martin Luther University Halle-Wittenberg, Am Kirchtor 1, 06108, Halle (Saale), Germany.

[2] German Centre for Integrative Biodiversity Research (iDiv) Halle-Jena-Leipzig, Puschstrasse 4, Leipzig 04103, Germany.

[3] Department of Animal and Plant Sciences, University of Sheffield. Western Bank, Sheffield S10 2TN, UK.

[4] Department of Physiological Diversity, Helmholtz-Centre for Environmental Research –UFZ, Permoserstrasse 15, Leipzig 04318, Germany.

[5] Departamento de Biologia – IVAGRO, Universidad de Cadiz, Campus Rio San Pedro, 11510 Puerto Real, Spain.

[6] Interdisciplinary Centre on Population Dynamics (CPop), University of Southern Denmark. Campusvej 55, 5230 Odense M, Denmark.

[7] Department of Biology, University of Southern Denmark. Campusvej 55, 5230 Odense M, Denmark.

[8] Department of Biology, Case Western Reserve University, Cleveland, OH 44106-7080.

[9] Alexander Center for Applied Population Biology, Conservation & Science Department, Lincoln Park Zoo, Chicago, IL 60614-4712 USA.

[10] Smithsonian Tropical Research Institute, Apartado 0843-03092, Balboa, Ancón, Panama.

[11] Department of Economics, University of Leipzig, Grimmaische Straße 12, 04109 Leipzig, Germany.

[12] Univ. Grenoble Alpes, INRAE, UR LESSEM, 38000 Grenoble, France.

[13] Centre for Applied Water Science, Institute for Applied Ecology, The University of Canberra, Canberra, Australian Capital Territory, Australia.

[14] Centre for Ecology and Conservation, College of Life and Environmental Sciences, University of Exeter, Penryn, United Kingdom.

[15] Institute of Evolutionary Ecology and Conservation Genomics, University of Ulm, Albert-Einstein-Allee 11, 89081, Ulm, Germany.

[16] Department of Zoology, University of Oxford. 11a Mansfield Road, Oxford, OX1 3SZ, United Kingdom.

[17] Department of Community Ecology, Helmholtz Centre for Environmental Research–UFZ, 06120 Halle (Saale), Germany.

* Corresponding author contact information:

Aldo Compagnoni

Phone: +49 341 9739143

Fax: +49 341 9739350

Email: aldo.compagnoni@idiv.de

German Centre for Integrative Biodiversity Research (iDiv)

Puschstrasse 4

04103 Leipzig Germany.

Supplementary Methods

Boolean expression used for the literature review.

We identified the studies for our review our literature review using the following Boolean expression:

(TS = (plant OR tree OR shrub OR herb OR herbaceous OR annual OR forb) AND TS = ("demographic model*" OR "projection matri*" OR “matrix population model*” OR MPM* OR "integral projection model*" OR IPM OR "stage-structured model*" OR “life table response experiment*” OR LTRE OR “population growth rate*”) AND TS = (environment* OR abiotic OR climat* OR “climate change” OR weather OR temperature OR precipitation OR rain* OR snow OR drought OR flood* OR warming OR disturbance) AND PY = (1997-2017).

Creation of density-independent matrix population models from the data by Chu et al. (2016)

We used data published by Chu *et al.* (2016)[1] to produce density-independent MPMs. To produce these MPMs, we fit statistical models as similar as possible to those used by Chu *et al.* (2016)[1]. The main difference is that our models did not include density-dependent terms. Below, we provide a brief explanation of the dataset, model fitting, and matrix projection construction.

The data from Chu *et al.* (2016)[1] provide information on the survival, growth, and recruitment of 12 species across five study sites. These study sites are located in Arizona, Idaho, Kansas, Montana, and New Mexico. Each site contains a set of one-square-meter plots subdivided in groups of plots that are spatially adjacent. Each plot was censused annually using a pantograph [2] that records the shape and position of each individual, classified as either adult or recruit. From the information on the shape of individuals, it is straightforward to infer the area of individuals; specifically, basal area for the grasses, and canopy area for the shrubs. These charts were digitized as shapefiles, and made public in a series of separate data articles[3–6]. These data are most commonly used to estimate the survival and growth of individuals, and the plot-level recruitment of seedlings[7–9]. The censuses extend from a minimum of 14 years at the Montana site, to a maximum of 35 years at the Kansas site.

We used these data to parameterize density-independent MPMs by fitting generalized linear mixed models to the survival, growth, and recruitment data. These models follow closely those fit by Dalgleish et al.[8] on the subset of data from Idaho [4]. In the individual models on survival and growth, we used the natural logarithm of basal or canopy area as our measure of individual size (henceforth *log(size)*). These models included random intercepts of year and group, and a random year slope of *log(size)*.

As reported elsewhere[9], in these data, model selection supports model fits with both a random intercept, and a random slope of *log(size)*. We fit the survival model assuming a Bernoulli distributed response. Our survival model thus had the form

$$s_{gyi} \sim Bernoulli(\hat{s}_{gyi}), \qquad \text{(S1a)}$$

$$logit(\hat{s}_{gyi}) = \alpha_y + \gamma_g + \beta_y \log(size)_i, \qquad \text{(S1b)}$$

$$\alpha_y \sim Normal(\hat{\alpha}, \sigma_\alpha), \qquad \text{(S1c)}$$

$$\gamma_g \sim Normal(\hat{\gamma}, \sigma_\gamma), \qquad \text{(S1d)}$$

$$\beta_y \sim Normal(\hat{\beta}, \sigma_\beta), \qquad \text{(S1e)}$$

where *g* refers to group, *y* refers to year, *i* refers to individual observations, α_y is the random effect of year, γ_g is the random effect of group, β_y is the random year effect of size, and $\hat{s}_{gyi}$ is predicted survival for each data point to time *y*. Here, note that $log(size)_i$ refers to time *y-1*, so that *log(size)* of individual *i* in year *y-1* is used to predict the average survival ($\hat{s}_{gyi}$) of individual *i* to year *y*.

The growth model had an identical structure, but it was a linear mixed effect model with normal response, so that:

$$log(size)_{gyi} \sim Normal(\hat{g}_{gyi}, \sigma_\varepsilon), \qquad \text{(S2a)}$$

$$\hat{g}_{gyi} = \alpha_y + \gamma_g + \beta_y \log(size)_i, \qquad \text{(S2b)}$$

$$\alpha_y \sim Normal(\hat{\alpha}, \sigma_\alpha), \qquad \text{(S2c)}$$

$$\gamma_g \sim Normal(\hat{\gamma}, \sigma_\gamma), \qquad \text{(S2d)}$$

$$\beta_y \sim Normal(\hat{\beta}, \sigma_\beta), \qquad \text{(S2e)}$$

where $\hat{g}_{gyi}$ is the predicted *log(size)* at time *y*, $log(size)_i$ refers to time *y-1*, σ_ε is the residual standard deviation of the normally distributed variable, and all other symbols reflect the same model structure as found in the survival model (Eq. S2). In these data, the residual variance is not constant but depends on size. We modeled this size

dependence on the residuals, defined as $r_{gyi} = log(size)_{gyi} - \hat{g}_{gyi}$, with the nonlinear function

$$r_{gyi} \sim ae^{b\hat{g}_{gyi}}, \qquad \text{(S3)}$$

which models the residual of observation of site *g*, year *y*, and individual *i* as a nonlinear function of the corresponding predicted size, $\hat{g}_{gyi}$, and parameters *a* and *b*. We fit the survival models in Eq. S1 using function glmer, the growth models in Eq. S2 using function lmer, both from the R package lme4[10], and we fit Eq. S3 using the R function nls[11].

Because recruitment data were available at the plot level, we used a different model structure. We used two predictors of plot-level recruitment: the plot-level and group-level cover of parents, following the model in Dalgleish et al. (2011)[8]. This model assumes that the number of recruits in year *y* and plot *i* reflects the cover of parents in the same plot *i* in year *y-1*, and dispersal from outside the plot, which should be proportional to the average cover of parents within the group of plots to which plot *i* belongs. Hence, we modeled recruitment of plot *i* in year *y* based on the cover of adults in the previous year, cov_i, and the average cover of adults in the group of plots to which plot *i* belongs, cov_g. We also modeled recruitment based on random effects of year and population. We modeled recruitment as a negative binomial process with a log link,

$$\lambda_{gyi} \sim NegBin(\hat{\lambda}_{gyi}, \theta), \qquad \text{(S4a)}$$

$$log(\hat{\lambda}_{gyi}) = cov_{true}\, e^{\,\beta_0+\alpha_y+\gamma_g}, \qquad \text{(S4b)}$$

$$cov_{true} = cov_i u + cov_g(1-u), \qquad \text{(S4c)}$$

$$\alpha_y \sim Normal(\hat{\alpha}, \sigma_\alpha), \qquad \text{(S4d)}$$

$$\gamma_g \sim Normal(\hat{\gamma_s}, \sigma_\gamma), \qquad \text{(S4e)}$$

where θ is the overdispersion parameter, $\hat{\lambda}_{gyi}$ is the average predicted number of recruits in plot *i*, group *g*, and year *y*, *u* is a parameter bounded between 0 and 1 that quantifies the contribution of the cover from each individual plot *i*, and the average cover of the group *g*, to the number of recruits of the following year, α_y is a random effect of year *y*, γ_g is a random effect of group *g*, and β_0 is the fixed effect of true cover.

We fit this model in a Bayesian framework, using Gibbs sampling via the R package R2jags[12]. We ran three chains for 25,000 iterations, with 5,000 iterations of burn in. We assumed parameters had converged if the Gelman-Rubin metric $\hat{R}$ was below 1.1. Finally, we checked our models performing posterior predictive checks[13].

We used these models to parameterize MPMs for each group and year. We created 10×10 MPMs, as suggested by Salguero-Gómez & Plotkin (2010)[14]. Our approach was similar to the construction of an IPM, in that we populated our MPMs using the parameters of the mixed effect models. However, IPMs usually model the size distribution of seedlings using a normal distribution. We did not use this method, because it would unintentionally evict most seedlings[15]. Instead, we placed all recruits in the smallest size class.

Methods for computing generation time

We calculated generation time (T) using methods described in[16]. We calculated generation time (T) by applying the age-specific survivorship and fertility curves

$$T = \frac{\Sigma(\mathrm{l}_x \mathrm{m}_x)\mathrm{x}}{\Sigma(\mathrm{l}_x \mathrm{m}_x)} \quad \text{(S6)}$$

where l_x and m_x are, respectively, survivorship and fertility at age x. These two age-specific functions were obtained using age-from-stage methods[17,18]. The vectors l_x and m_x were trimmed at the quasi-stable stage of 95% convergence to the stable stage distribution to avoid potential spurious plateaus of mortality and fertility [19] as described in Jones et al. (2014)[20].

Re-running the tests of hypothesis 2 and 4 excluding graminoid species

Re-running our tests of H_2 and H_4 with a reduced dataset, further attenuated the results referred to H_2, and upheld the results for H_4. When testing H_2 using the full dataset, 90.5% of our bootstrap samples had slopes below zero (β_{meta} = -3.83 x 10^{-5}, 95% C.I. [-9.47 x 10^{-5}, 1.99 x 10^{-5}]). However, when using the reduced dataset, the bootstrap samples below zero fell to 74%, and the β_{meta} values were attenuated (β_{meta} = -1.92 x 10^{-5}, 95% C.I. [-7.92 x 10^{-5}, 4.32 x 10^{-5}]). When testing H_4 using the full dataset, 100% of simulated β_{meta} values referring to the effect of precipitation were below zero (β_{meta} = -0.54, 95% C.I. [-0.63, -0.44]). Fitting this meta-regression using the reduced dataset provided qualitatively identical results. The percentage of simulated β_{meta} values below zero was 100%, and β_{meta} values were similar (β_{meta} = -0.55, 95% C.I. [-0.75, -0.34]).

Supplementary Figures

Supplementary Figure 1. Geographic and locations and position in a Whittaker biome plot (insert) of the plant demographic studies used in this manuscript. The background grey scale represents[21] terrestrial biomes. These shapefiles used to produce this map are provided by The Nature Conservancy at https://doi.org/10.7910/DVN/WTLNRG.

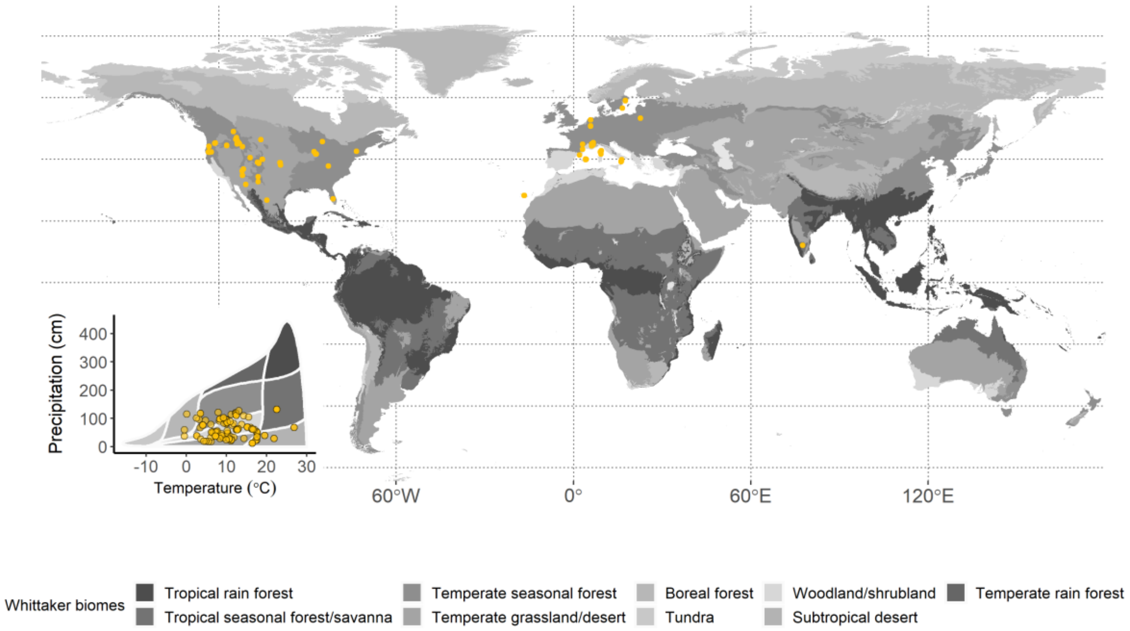

Supplementary Figure 2. A) Relationship between the standard deviation in annual precipitation and water availability index (WAI), and B) between the standard deviation of annual temperature, and mean annual temperature (MAT). The standard deviations are calculated over a 40-year period of annual data, as detailed in the main manuscript. Each point refers to one of the 162 plant populations included in our study. In each plot, the thick black line is the mean prediction of the linear model describing the relationship. The shaded area is the 95% confidence interval around the mean prediction.

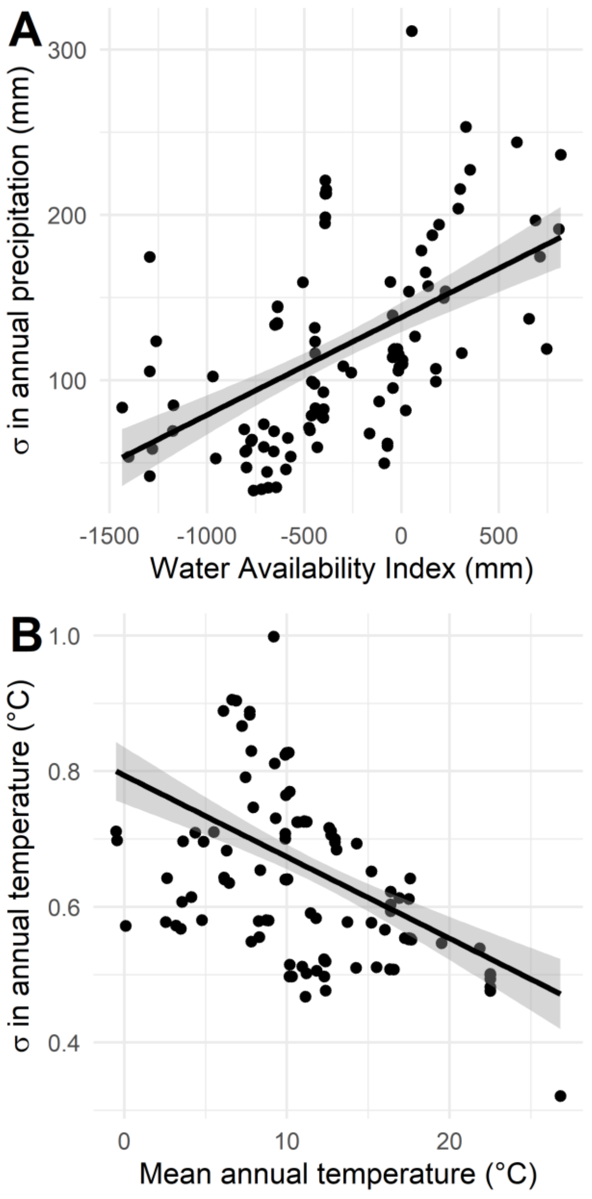

Supplementary Figure 3. Nonlinear relationship between log population growth rate (λ) and temperature anomalies. The plots refer to the four plant populations for which this nonlinear relationship had the highest AICc weight compared to the remaining five competing models (Supplementary Table 1). In each plot, the thick blue line is the mean prediction of the nonlinear model. The shaded area is the 95% confidence interval around the mean prediction.

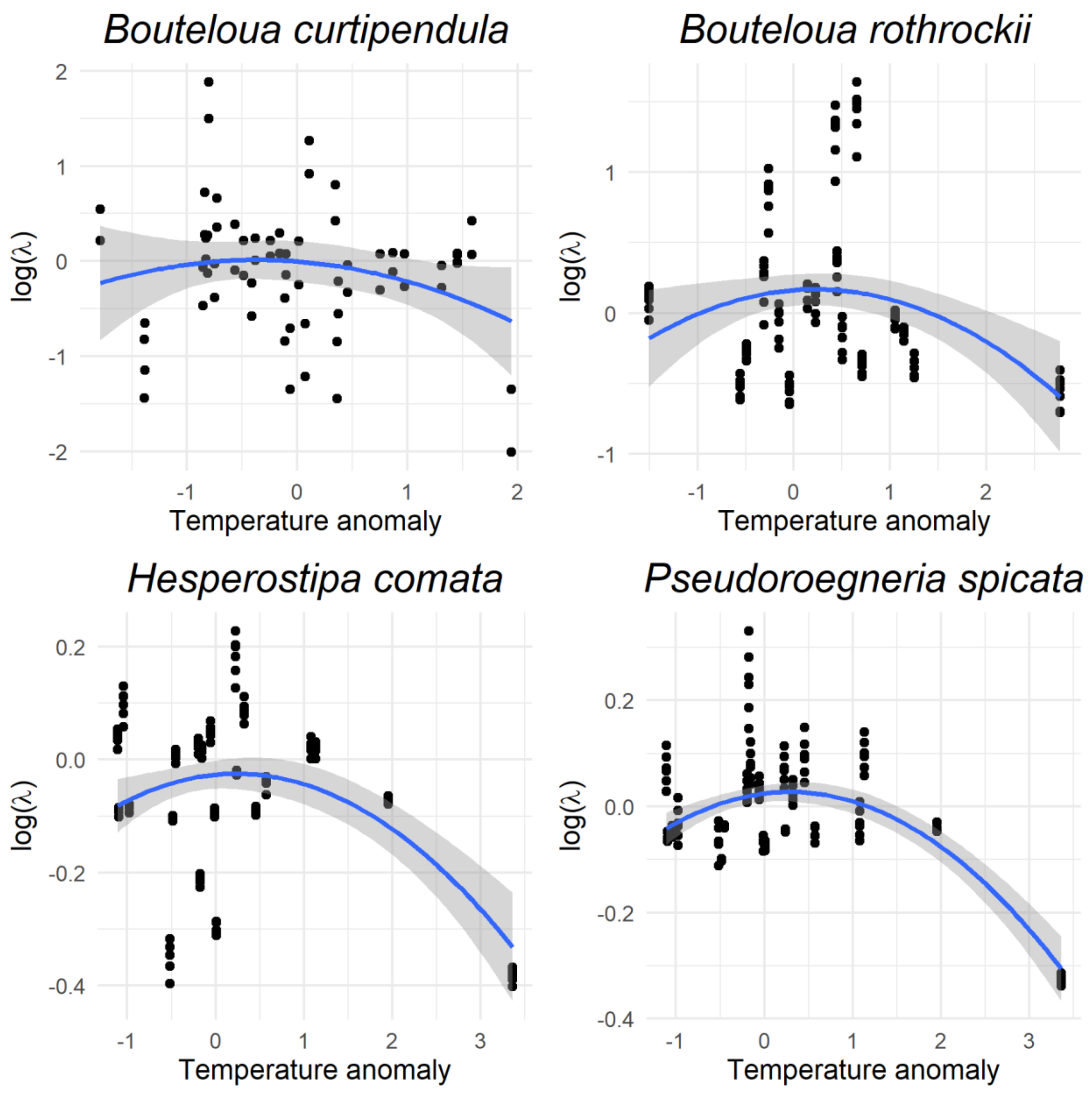

Supplementary Figure 4. Nonlinear relationship between log population growth rate (λ) and precipitation anomalies. The plots refer to the two plant populations for which this nonlinear relationship had the highest AICc weight compared to the remaining five competing models (Supplementary Table 1). In each plot, the thick blue line is the mean prediction of the nonlinear model. The shaded area is the 95% confidence interval around the mean prediction.

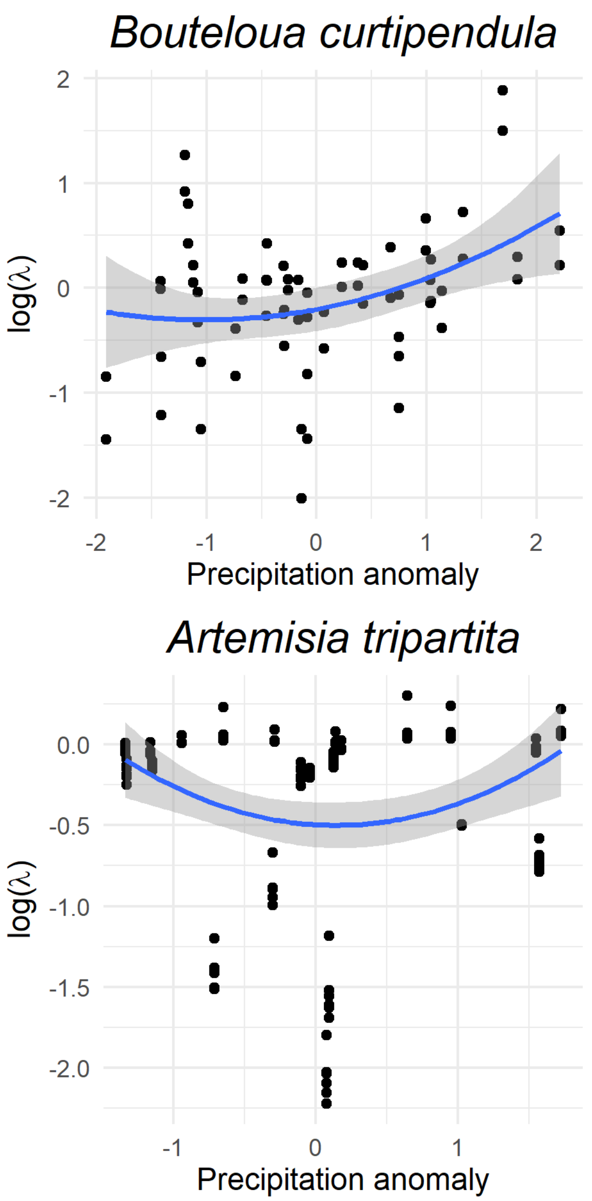

Supplementary Figure 5. Nonlinear relationship between log population growth rate (λ) and both precipitation and temperature anomalies. The plots refer to the four plant populations for which this nonlinear relationship had the highest AICc weight compared to the remaining five competing models (Supplementary Table 1). In each plot, the thick blue line is the mean prediction of the nonlinear model. The shaded area is the 95% confidence interval around the mean prediction.

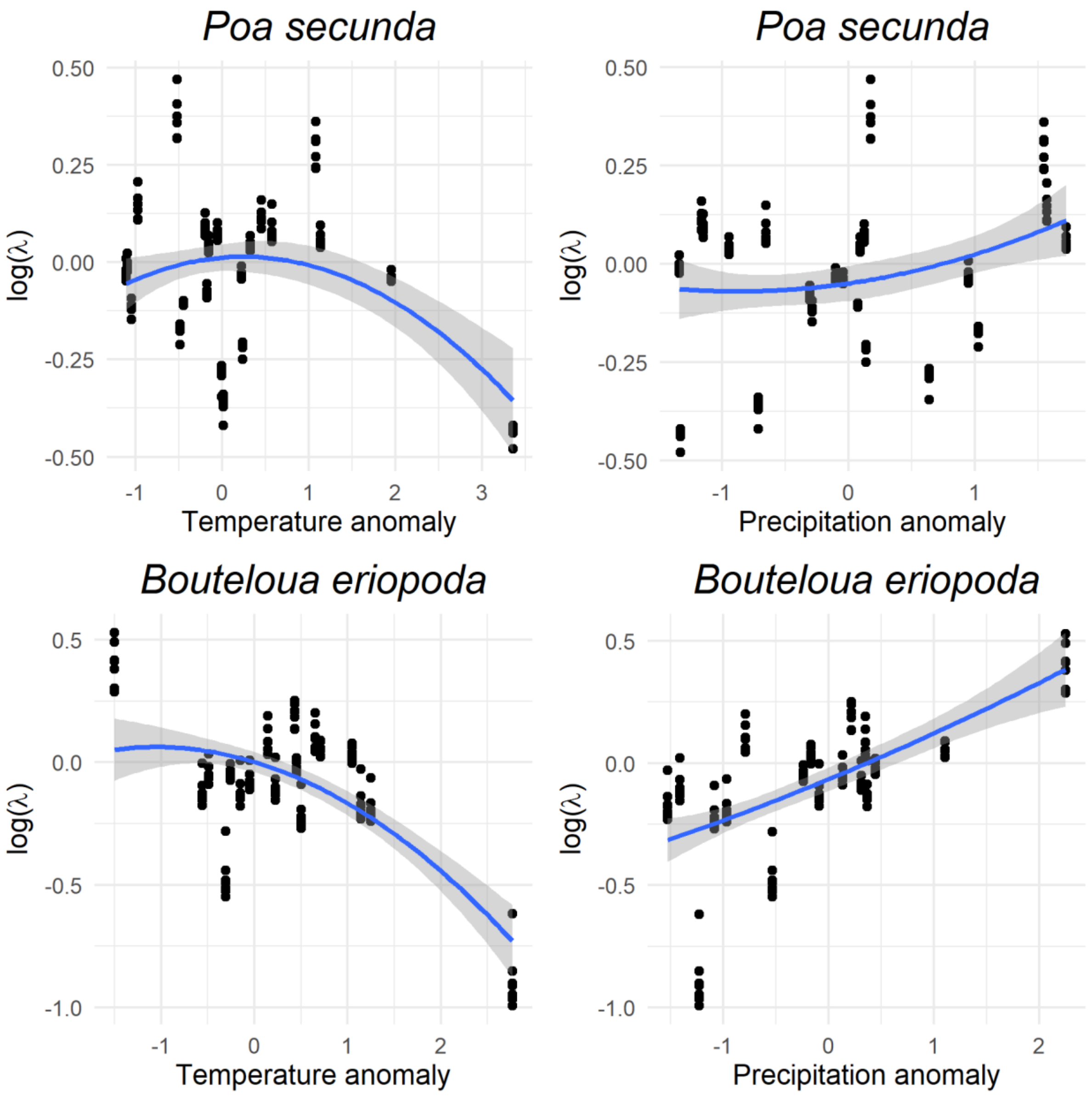

Supplementary Figure 6. Effect sizes of precipitation (A) and temperature (B) based on five plant types. The boxplots hinges refer to the 25th and 75th percentiles of the data. The whiskers extend 1.5 the interquartile range beyond the hinges. The think black line in the middle of the boxplots is the median. Circles show the raw effect sizes, and they are jittered along the x-axis. The sample sizes are denoted by the symbol N, which refers to the number of populations analyzed.

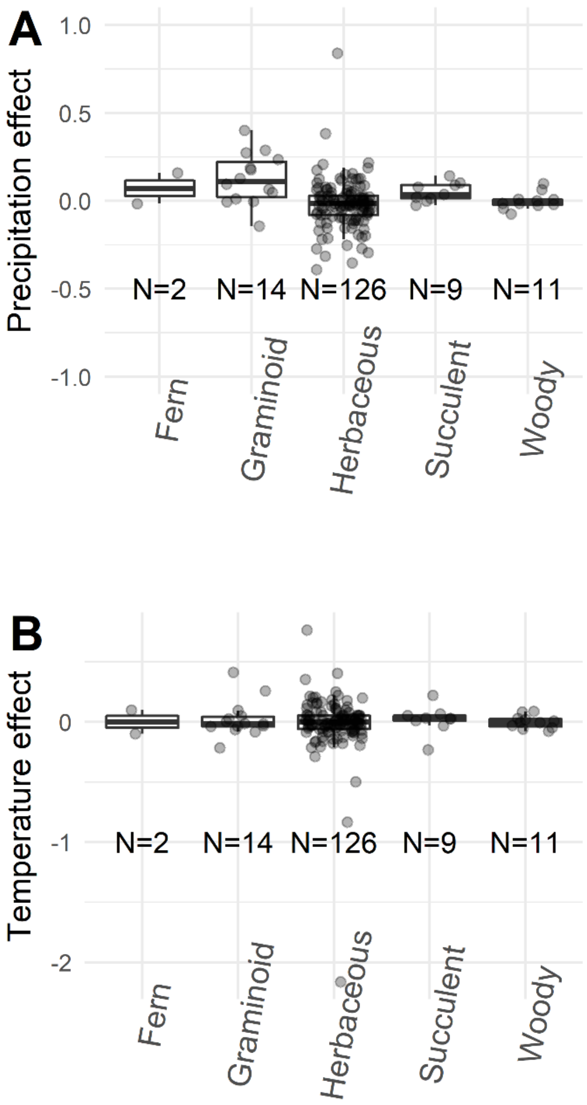

Supplementary Figure 7. Reanalysis of hypotheses 2 after removing graminoid species. Effect of precipitation anomalies on the logged asymptotic population growth rate (λ) as a function of water availability index when graminoids are excluded from the dataset. This figure reproduces Figure 1A in the main text. The y-axis represents the effect sizes of yearly anomalies in precipitation. The uncertainty of these effect sizes is shown by the size of circles, which are inversely proportional to the standard error (SE) of effect sizes (1/SE). The thick black line shows the mean prediction of the meta-regression; this line is dashed because the relationship is non-significant. The shaded areas represent the 95% confidence interval of 1000 bootstrapped linear regressions. Dashed lines show the zero effect. The color of individual data points shows four separate plant types.

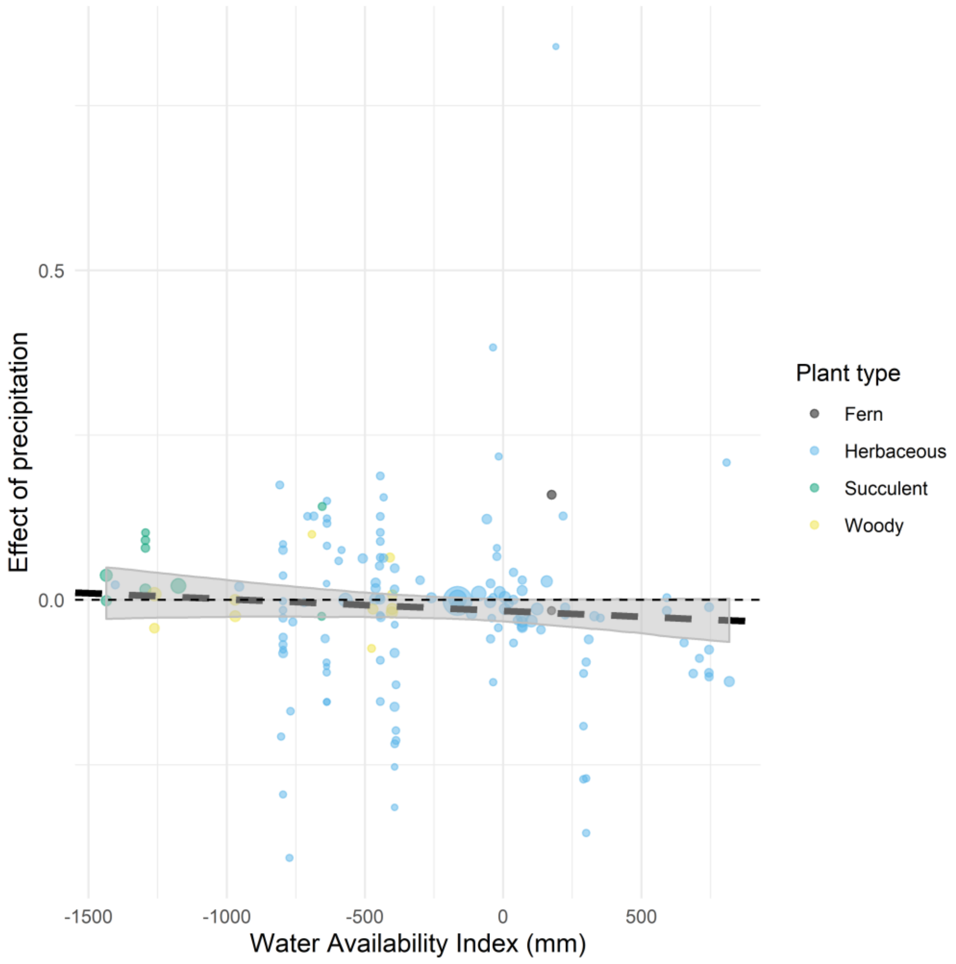

Supplementary Figure 8. Reanalysis of hypotheses 4 after removing graminoid species. The absolute effect of precipitation as a function of logged generation time (*T*) when graminoids are excluded from the dataset. This figure reproduces Figure 2A in the main text. We show the effect sizes of precipitation as a function of log(*T*). The uncertainty of these effect sizes is shown by the size of circles, which are inversely proportional to the standard error (SE) of effect sizes (1/SE). The thick black line shows the mean prediction of the meta-regression. The shaded areas represent the 95% confidence interval of 1000 bootstrapped gamma regressions. The color of individual data points shows four separate plant types.

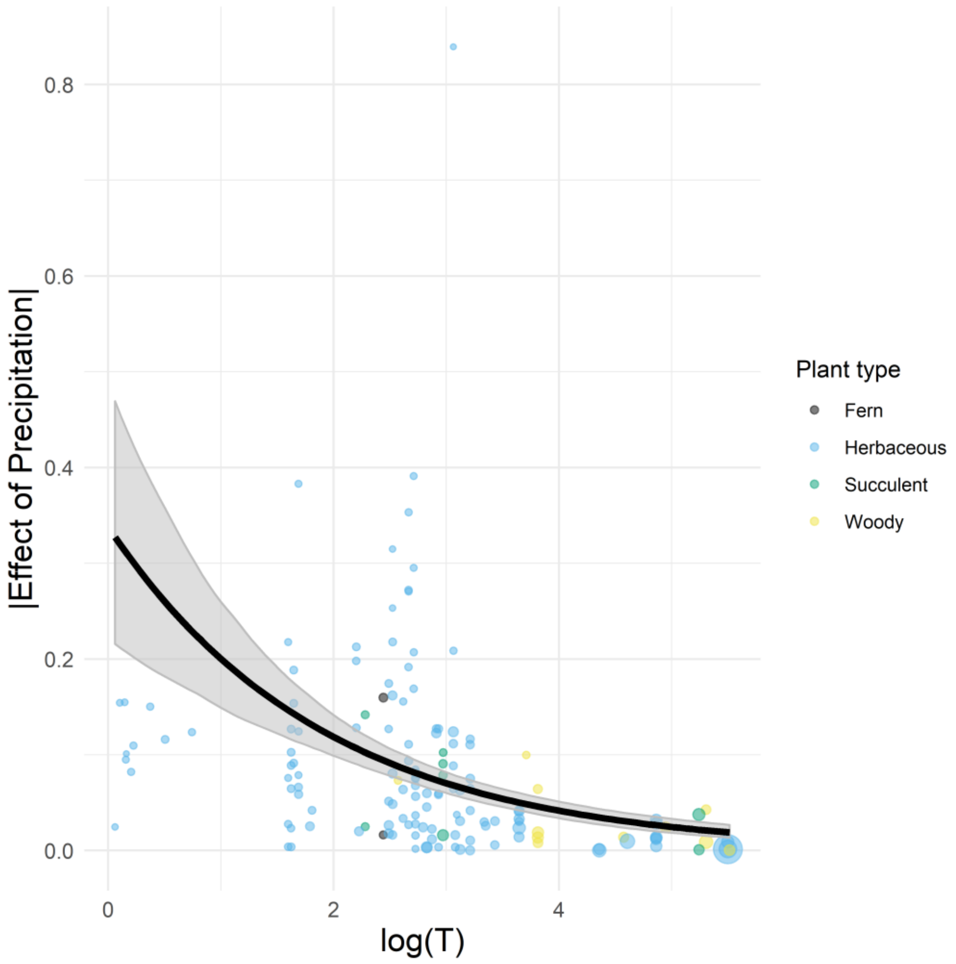

Supplementary Tables

Supplementary Table 1. Statistical models tested to estimate the effect of climate, and potential interactions between climatic and non-climatic covariates, on population growth rate.

Model formula	Description
$log(\lambda)_y = \alpha + \beta_p P_y + \beta_t T_y + \varepsilon_y$	Baseline model
$log(\lambda)_y = \alpha + \beta_p P_y + \beta_t T_y + \beta_{t2} T_y^2 + \varepsilon_y$	Quadratic effect of temperature
$log(\lambda)_y = \alpha + \beta_p P_y + \beta_t T_y + \beta_{p2} P_y^2 + \varepsilon_y$	Quadratic effect of precipitation
$log(\lambda)_y = \alpha + \beta_p P_y + \beta_t T_y + \beta_{p2} P_y^2 + \beta_{t2} T_y^2 + \varepsilon_y$	Quadratic effect of both climate factors
$log(\lambda)_y = \alpha + \beta_p P_y + \beta_t T_y + \beta_c C_i + \beta_x P_y x C_i + \varepsilon_y$	Covariate and precipitation interaction
$log(\lambda)_y = \alpha + \beta_p P_y + \beta_t T_y + \beta_c C_i + \beta_x T_y x C_i + \varepsilon_y$	Covariate and temperature interaction

Supplementary Table 2. Results of the Tukey's Honest Significant Difference test comparing the mean effect sizes of precipitation among five plant types. This is a two-sided test.

Comparison	Difference	Adjusted p-value
Graminoid-Fern	0.054087	0.983293
Herbaceous-Fern	-0.08947	0.879586
Succulent-Fern	-0.02028	0.999675
Woody-Fern	-0.07216	0.955062
Herbaceous-Graminoid	-0.14355	0.001723
Succulent-Graminoid	-0.07436	0.686943
Woody-Graminoid	-0.12624	0.133784
Succulent-Herbaceous	0.069191	0.559833
Woody-Herbaceous	0.017311	0.993808
Woody-Succulent	-0.05188	0.908479

Supplementary Table 3. Results of the Tukey's Honest Significant Difference test comparing the mean effect size of temperature among five plant types. This is a two-sided test.

**Comparison**	**Difference**	**Adjusted p-value**
Graminoid-Fern	0.024553	0.999906
Herbaceous-Fern	-0.01376	0.999988
Succulent-Fern	0.019234	0.999969
Woody-Fern	0.000325	1
Herbaceous-Graminoid	-0.03832	0.975321
Succulent-Graminoid	-0.00532	0.999998
Woody-Graminoid	-0.02423	0.998921
Succulent-Herbaceous	0.032996	0.993436
Woody-Herbaceous	0.014087	0.999662
Woody-Succulent	-0.01891	0.999737

Supplementary References

1. Chu, C. *et al.* Direct effects dominate responses to climate perturbations in grassland plant communities. *Nat. Commun.* **7**, 11766 (2016).
2. Hill, R. R. Charting Quadrats with a Pantograph. *Ecology* **1**, 270–273 (1920).
3. Adler, P. B., Tyburczy, W. R. & Lauenroth, W. K. Long-term mapped quadrats from Kansas prairie: demographic information for herbaceous plants. *Ecology* **88**, 2673–2673 (2007).
4. Zachmann, L., Moffet, C. & Adler, P. Mapped quadrats in sagebrush steppe: long-term data for analyzing demographic rates and plant–plant interactions. *Ecology* **91**, 3427–3427 (2010).
5. Anderson, J., Vermeire, L. & Adler, P. B. Fourteen years of mapped, permanent quadrats in a northern mixed prairie, USA. *Ecology* **92**, 1703–1703 (2011).
6. Anderson, J., McClaran, M. P. & Adler, P. B. Cover and density of semi-desert grassland plants in permanent quadrats mapped from 1915 to 1947. *Ecology* **93**, 1492–1492 (2012).
7. Adler, P. B., Ellner, S. P. & Levine, J. M. Coexistence of perennial plants: an embarrassment of niches. *Ecol. Lett.* **13**, 1019–1029 (2010).
8. Dalgleish, H. J., Koons, D. N., Hooten, M. B., Moffet, C. A. & Adler, P. B. Climate influences the demography of three dominant sagebrush steppe plants. *Ecology* **92**, 75–85 (2011).
9. Tredennick, A. T., Teller, B. J., Adler, P. B., Hooker, G. & Ellner, S. P. Size-by-environment interactions: a neglected dimension of species' responses to environmental variation. *Ecol. Lett.* **21**, 1757–1770 (2018).
10. Bates, D., Mächler, M., Bolker, B. & Walker, S. Fitting Linear Mixed-Effects Models Using lme4. *J. Stat. Softw.* **67**, 1–48 (2015).

11. R Core Team. *R: A language and environment for statistical computing*. (R Foundation for Statistical Computing, 2019).
12. Yu-Sung, S. & Yajima, M. *R2jags: Using R to Run 'JAGS'. R package version 0.5-7*. (2015).
13. Gelman, A. *et al. Bayesian Data Analysis, Third Edition*. (CRC Press, 2013).
14. Salguero-Gómez, R. & Plotkin, J. B. Matrix Dimensions Bias Demographic Inferences: Implications for Comparative Plant Demography. *Am. Nat.* **176**, 710–722 (2010).
15. Williams, J. L., Miller, T. E. X. & Ellner, S. P. Avoiding unintentional eviction from integral projection models. *Ecology* **93**, 2008–2014 (2012).
16. Salguero-Gómez, R. *et al.* Fast–slow continuum and reproductive strategies structure plant life-history variation worldwide. *Proc. Natl. Acad. Sci.* **113**, 230–235 (2016).
17. Cochran, M. E. & Ellner, S. Simple methods for calculating age-based life history parameters for stage-structured populations. *Ecol. Monogr.* **62**, 345–364 (1992).
18. Caswell, H. *Matrix population models.* (Massachusetts: Sinauer Associates, 2001).
19. Horvitz, C. C. & Tuljapurkar, S. Stage dynamics, period survival, and mortality plateaus. *Am. Nat.* **172**, 203–215 (2008).
20. Jones, O. R. *et al.* Diversity of ageing across the tree of life. *Nature* **505**, 169–173 (2014).
21. Whittaker, R. H. *Communities and Ecosystems*. (Macmillan, London: Collier Macmillan, 1970).
