# Supplementary material for: Herbaceous perennial plants with short generation time have stronger responses to climate anomalies than those with longer generation time
Source: Nat Commun. 2021 Mar 23;12:1824. doi: 10.1038/s41467-021-21977-9 (PMC7988175; doi:10.1038/s41467-021-21977-9)
Supplement: Supplementary file 3 — Description of Additional Supplementary Files [file 41467_2021_21977_MOESM3_ESM.docx]

Description of Additional Supplementary Files

**File Name: Supplementary Data 1**

**Description:** List of studies, and associated plant species, providing the Matrix Population Models (MPMs) or Integral Projection Models (IPMs) used in this manuscript. We provide information on the originating article, the spatio-temporal replication of the study, the inclusion in the COMPADRE (v. 5.0.1; <https://compadre-db.org/Data/Compadre>) and PADRINO (beta version; <https://github.com/levisc8/rpadrino>) databases, and the type of non-climatic covariates linked to each population model. We used these archived projection models in our comparative analyses.
