# Supplementary material for: Herbaceous perennial plants with short generation time have stronger responses to climate anomalies than those with longer generation time
Source: Nat Commun. 2021 Mar 23;12:1824. doi: 10.1038/s41467-021-21977-9 (PMC7988175; doi:10.1038/s41467-021-21977-9)
Supplement: Supplementary file 5 — Reporting Summary [file 41467_2021_21977_MOESM5_ESM.pdf]

nature research

Corresponding author(s): Aldo Compagnoni

Last updated by author(s): Feb 7, 2021

Reporting Summary

Nature Research wishes to improve the reproducibility of the work that we publish. This form provides structure for consistency and transparency in reporting. For further information on Nature Research policies, see our Editorial Policies and the Editorial Policy Checklist.

Statistics

For all statistical analyses, confirm that the following items are present in the figure legend, table legend, main text, or Methods section.

n/a	Confirmed	
☐	☒	The exact sample size (*n*) for each experimental group/condition, given as a discrete number and unit of measurement
☐	☒	A statement on whether measurements were taken from distinct samples or whether the same sample was measured repeatedly
☐	☒	The statistical test(s) used AND whether they are one- or two-sided<br>*Only common tests should be described solely by name; describe more complex techniques in the Methods section.*
☐	☒	A description of all covariates tested
☐	☒	A description of any assumptions or corrections, such as tests of normality and adjustment for multiple comparisons
☐	☒	A full description of the statistical parameters including central tendency (e.g. means) or other basic estimates (e.g. regression coefficient) AND variation (e.g. standard deviation) or associated estimates of uncertainty (e.g. confidence intervals)
☐	☒	For null hypothesis testing, the test statistic (e.g. F, t, r) with confidence intervals, effect sizes, degrees of freedom and P value noted<br>*Give P values as exact values whenever suitable.*
☒	☐	For Bayesian analysis, information on the choice of priors and Markov chain Monte Carlo settings
☐	☒	For hierarchical and complex designs, identification of the appropriate level for tests and full reporting of outcomes
☐	☒	Estimates of effect sizes (e.g. Cohen's *d*, Pearson's *r*), indicating how they were calculated

Our web collection on statistics for biologists contains articles on many of the points above.

Software and code

Policy information about availability of computer code

Data collection	R version 3.6.1
Data analysis	R version 3.6.1

For manuscripts utilizing custom algorithms or software that are central to the research but not yet described in published literature, software must be made available to editors and reviewers. We strongly encourage code deposition in a community repository (e.g. GitHub). See the Nature Research guidelines for submitting code & software for further information.

Data

Policy information about availability of data

All manuscripts must include a data availability statement. This statement should provide the following information, where applicable:

- Accession codes, unique identifiers, or web links for publicly available datasets
- A list of figures that have associated raw data
- A description of any restrictions on data availability

The raw data is archived in the COMPADRE Database 5.0.0, PADRINO, and CHELSA databases. The formatted dataset, metadata, and code for final analyses is now publicly accessible on Github at http://doi.org/10.5281/zenodo.4516446

Field-specific reporting

Ecological, evolutionary & environmental sciences study design

All studies must disclose on these points even when the disclosure is negative.

Study description	We examine whether water availability, mean annual temperature, and species life history can predict how strongly plant population growth rates respond to annual precipitation and temperature anomalies.
Research sample	Two data sources: the first data source was composed of time series of population growth rates derived from previously published plant population projection models (Matrix and Integral population projection models). We obtained matrix projection models selecting them from COMPADRE, version 5.0.1: https://compadre-db.org/Data/Compadre. We obtained integral projection models from PADRINO, beta version: https://github.com/levisc8/rpadrino. The second data source was gridded climatic data referred to the locations and times referred to the time series of population growth rates of point 1. We obtained these data from the CHELSAcruts database at http://dx.doi.org/10.16904/envidat.159.
Sampling strategy	We gathered all available time series of annual population growth rates longer than 5 years derived from published population projection models (matrix and integral projection models).
Data collection	Regarding time series of population growth rates, we extracted already public data from the COMPADRE open-access database (version 5.0.0), or digitized plant population projection models into COMPADRE or PARDINO (a new, currently in development, open-access database on Integral Projection Models) when these contained more than 5 annual population growth rates. Regarding gridded climatic data, we used the publicly available CHELSA database.
Timing and spatial scale	Our datasets comprise data from 1915 to 2016. Many of these time series are continuous, but some have gaps due to circumstances occurred during field sampling (mostly funding). Our demographic datasets have a spatial scale of a few squared meters, because plant populations are monitored tracking a few (from a minimum of one to a maximum of about 30) one-square meter plots. On the other hand, the gridded climatic data we used from CHELSA has a 1Km2 resolution.
Data exclusions	We excluded time series of population growth rates shorter than 6 years. We made this choice subjectively to balance precision of our estimates, with the total sample size of our synthesis.
Reproducibility	We provide our data and code at the github repository at DOI http://doi.org/10.5281/zenodo.4516446. This repository allows reproducing all of our main results.
Randomization	We have used all available data in the literature to carry out a synthesis of existing knowledge. Because this sample is biased, we addressed such bias in the analyses (namely, the analysis on the role of taxonomic bias), and we addressed the role of both geographic and taxonomic biases in the discussion of the article.
Blinding	Blinding is not relevant to our study, because our time series of population abundance are long (up to 33 years in our datasets), and many datasets were collected in either monitoring projects, or to test hypotheses disconnected from climate effects. We therefore expect sampling bias to be non-existent or low.

Did the study involve field work? ☐ Yes ☒ No

Reporting for specific materials, systems and methods

We require information from authors about some types of materials, experimental systems and methods used in many studies. Here, indicate whether each material, system or method listed is relevant to your study. If you are not sure if a list item applies to your research, read the appropriate section before selecting a response.

Materials & experimental systems

n/a	Involved in the study
☒	☐ Antibodies
☒	☐ Eukaryotic cell lines
☒	☐ Palaeontology and archaeology
☒	☐ Animals and other organisms
☒	☐ Human research participants
☒	☐ Clinical data
☒	☐ Dual use research of concern

Methods

n/a	Involved in the study
☒	☐ ChIP-seq
☒	☐ Flow cytometry
☒	☐ MRI-based neuroimaging
